# Novel Insight Into the Role of Immune Dysregulation in Amyotrophic Lateral Sclerosis Based on Bioinformatic Analysis

**DOI:** 10.3389/fnins.2021.657465

**Published:** 2021-04-30

**Authors:** Yongzhi Xie, Ximei Luo, Haiqing He, Min Tang

**Affiliations:** ^1^Department of Neurology, The Third Xiangya Hospital, Central South University, Changsha, China; ^2^School of Computer Science and Technology, Harbin Institute of Technology, Harbin, China; ^3^Department of Urology, The Second Xiangya Hospital, Central South University, Changsha, China; ^4^Department of Geriatrics, Third Xiangya Hospital, Central South University, Changsha, China

**Keywords:** amyotrophic lateral sclerosis, immune, bioinformatics, ssGSEA, WGCNA, LASSO

## Abstract

Amyotrophic lateral sclerosis (ALS) is a fatal neurodegenerative disorder characterized by the progressive degeneration of motor neurons. The causative pathogenic mechanisms in ALS remain unclear, limiting the development of treatment strategies. Neuroinflammation and immune dysregulation were involved in the disease onset and progression of several neurodegenerative disorders, including ALS. In this study, we carried out a bioinformatic analysis using publicly available datasets from Gene Expression Omnibus (GEO) to investigate the role of immune cells and genes alterations in ALS. Single-sample gene set enrichment analysis revealed that the infiltration of multiple types of immune cells, including macrophages, type-1/17 T helper cells, and activated CD4 + /CD8 + T cells, was higher in ALS patients than in controls. Weighted gene correlation network analysis identified immune genes associated with ALS. The Gene Ontology analysis revealed that receptor and cytokine activities were the most highly enriched terms. Pathway analysis showed that these genes were enriched not only in immune-related pathways, such as cytokine-cytokine receptor interaction, but also in PI3K-AKT and MAPK signaling pathways. Nineteen immune-related genes (*C3AR1*, *CCR1*, *CCR5*, *CD86*, *CYBB*, *FCGR2B*, *FCGR3A*, *HCK*, *ITGB2*, *PTPRC*, *TLR1*, *TLR2*, *TLR7*, *TLR8*, *TYROBP*, *VCAM1*, *CD14*, *CTSS*, and *FCER1G*) were identified as hub genes based on least absolute shrinkage and selection operator analysis. This gene signature could differentiate ALS patients from non-neurological controls (*p* < 0.001) and predict disease occurrence (AUC = 0.829 in training set; AUC = 0.862 in test set). In conclusion, our study provides potential biomarkers of ALS for disease diagnosis and therapeutic monitoring.

## Introduction

Amyotrophic lateral sclerosis (ALS) is a fatal neurodegenerative disorder predominantly affecting motor neurons. It has a prevalence of 4.42 per 100,000 people worldwide, which increases by age until the age of 70–79 (Logroscino et al., 2010; Ingre et al., 2015; Xu et al., 2020). As a devastating disease, ALS most frequently involves spinal and bulbar muscles but even respiratory muscles can be affected (Taylor et al., 2016; Brown and Al-Chalabi, 2017). ALS is essentially a sporadic disorder, with only ∼10% of cases involving a genetic link, and in most patients the etiology is unclear. The clinical heterogeneity of ALS makes diagnosis challenging, especially as no diagnostic tests are yet available (van Es et al., 2017). Moreover, the intervention strategies are limited; approved medications provide only modest benefits, and therapeutic approaches are largely supportive and involve symptom management primarily focused on the respiratory system (Petrov et al., 2017; Mejzini et al., 2019). ALS becomes progressively generalized and patients die about 3–4 years after disease onset (van Es et al., 2017). Detailed knowledge of ALS development can lead to more effective management at an early stage of disease.

Immune/inflammatory abnormalities are a common pathologic feature of many neurodegenerative disorders, and there is increasing evidence that immune dysregulation plays a critical role in ALS onset and progression (Morello et al., 2017). Human postmortem studies have revealed immune abnormalities at the end stage of ALS. Microglia activation is also a common pathologic feature of the disease (McGeer and McGeer, 2002; Shibata et al., 2009; McCauley and Baloh, 2019). The innate immune response, rather than adaptive immunity, is thought to mediate neuroinflammation in ALS. Infiltrating immune cells are also found in the central nervous system (CNS) in human ALS, including monocytes, macrophages, neutrophils, and T cells in motor neuron destruction (Graves et al., 2004; Lawson et al., 2008; Prinz and Priller, 2017; Beers and Appel, 2019). Systemic immune dysregulation has also been reported, as evidenced by elevated levels of inflammatory markers and altered circulating lymphocyte and monocyte populations (Zhang et al., 2005; McCombe and Henderson, 2011; Murdock et al., 2017). However, whether the immune response in ALS is protective or harmful is debated; clarifying the mechanisms of immune dysfunction in ALS may provide a basis for the development of a novel treatment.

In this study, we carried out a bioinformatic analysis using publicly available gene expression datasets to investigate the role of immune cell and expression of immune-related genes (IRGs) in ALS. We are trying to provide novel insights into the role of immune-related mechanisms in ALS and identify a signature that can be used to predict disease occurrence.

## Materials and Methods

### Data Collection and Processing

The Gene Expression Omnibus (GEO)^1^, an international repository of gene expression, is a free public database (Barrett et al., 2013). The GSE153960 dataset includes 1,838 samples (non-neurological control, ALS spectrum motor neuron disease [MND], other neurological disorders, other MND, and familial ALS) and mRNA expression data derived from postmortem tissue specimens of the cerebellum, cortex, spinal cord, and hippocampus (Prudencio et al., 2020). We extracted the expression matrix of non-neurological control and ALS-spectrum MND. The datasets consisted of two parts, one part was referred to as the primary dataset which was based on the platforms of GPL24676 (Illumina NovaSeq 6000), the other one was referred to as the secondary dataset and was based on the platforms of GPL16791 (Illumina HiSeq 2500). Data for 684 ALS spectrum NMD patients and 190 non-neurological controls from the primary dataset and 546 ALS spectrum NMD patients and 90 non-neurological controls from the secondary dataset were analyzed in our study. The raw count matrices were normalized and transformed into fragments per kilobase of sequence per million mapped reads (FPKM) values for further analysis. A total of 1,713 IRGs were downloaded from the ImmPort online database^2^.

### Evaluation of Immune Cell Infiltration

Single-sample gene set enrichment analysis (ssGSEA) was performed using the Gene Set Variation Analysis (GSVA) package in R (version 4.0.3) software^3^ to examine immune cell infiltration in ALS patients and non-neurological control subjects (Barbie et al., 2009; Hänzelmann et al., 2013). We focused on 12 types of immune cell that were shown to be associated with ALS in previous studies (Malaspina et al., 2015), including macrophages, regulatory T cells (Tregs), type-1 T helper cells (Th1), type-2 T helper cells (Th2), type-17 T helper cells (Th17), activated CD4 + T cells, activated CD8 + T cells, monocytes, activated dendritic cells, neutrophils, mast cells, and myeloid-derived suppressor cells. The detailed gene sets of each immune cell type were obtained from a previous study (Charoentong et al., 2017). ssGSEA scores representing the relative abundance of each immune cell type were calculated by ssGSEA analysis and normalized to unity distribution (with 0 and 1 as the minimum and maximum values, respectively). The Wilcoxon test was used to evaluate differences between ALS and non-neurological control groups. An adjusted *p* value < 0.05 (after Benjamini-Hochberg correction) was defined as statistically significant.

### Weighted Gene Correlation Network Analysis (WGCNA)

The expression profiles of all IRGs were extracted from the primary dataset and could be used for WGCNA analysis based on previous studies (Huang et al., 2020; Wang et al., 2020). To examine the relationship between immune gene and phenotype (i.e., ALS or non-neurological control), we generated unsigned co-expression networks using the WGCNA package in R (Langfelder and Horvath, 2008). Briefly, the log2 (FPKM + 1) transformed data were used to calculate Pearson’s correlation matrices. For module construction, the soft thresholding power β (1 to 20) was screened with the integrated *pickSoftThreshold* function. A suitable power β was selected to increase co-expression similarity and achieve scale-free topology. Co-expression modules were constructed with the minimum size set to 30. IRGs with similar expression patterns were grouped into modules. We assessed the correlation between phenotype and each module by Pearson’s correlation analysis and identified ALS-related modules; the genes in these modules were considered as ALS-related IRGs.

### Gene Ontology (GO) and Kyoto Encyclopedia of Genes and Genomes (KEGG) Pathway Analyses

Gene ontology and Kyoto encyclopedia of genes and genomes pathway analyses of genes in each identified module were carried out using *clusterProfiler* R package (Yu et al., 2012) to determine the biological functions of the genes and associated pathways. We adjusted the *p* value with the Benjamini-Hochberg method for multiple comparisons, and p.adjust < 0.05 was set as the cutoff.

### Protein-Protein Interaction (PPI) Network Analysis

We screened differentially expressed IRGs in the primary dataset (raw counts) using DESeq2 R package (Love et al., 2014). The cut-off criteria of adjusted *p* < 0.05 and | log_2_(fold change)| > 0.5 were considered as statistically significant. Overlapping genes between differentially expressed and ALS-related IRGs identified by WGCNA were selected as candidates. To analyze their interactions, we constructed a PPI network using the Search Tool for the Retrieval of Interacting Genes (STRING) online database^4^ (Szklarczyk et al., 2019). We then visualized the PPI network with Cytoscape v3.7.2 software and used the Cytohubba plugin to calculate the maximal clique centrality (MCC) degree of each node (gene) (Shannon et al., 2003). The top 25 genes with the highest MCC score were retained for further analysis.

### Least Absolute Shrinkage and Selection Operator (LASSO) Analysis

Least absolute shrinkage and selection operator analysis has strong predictive value and can prevent model overfitting (Tibshirani, 1996). To distinguish ALS patients from non-neurological control subjects, we constructed a LASSO model with the top 25 genes expression profiles using the glmnet package in R (binomial Lasso)^5^. The immune-score of this model for each sample was calculated with the equation immune-score = Σexpgenei^∗^ βi, where expgenei is the relative expression of the gene in the signature for patient i, and βi is the regression coefficient of gene i from the LASSO analysis.

### Validation of LASSO Model

We randomly divided the primary dataset into the training (70%, *N* = 614) and test (30%, *N* = 260) sets. Patients in these sets constituted the training and internal validation cohort, respectively, while those in the secondary dataset (*N* = 636) served as the external validation cohort. To evaluate the performance of the LASSO model for predicting the occurrence of ALS, we performed receiver operating characteristic (ROC) curve analyses for the three cohorts using the pROC package of R (Robin et al., 2011).

## Results

### Immune Cell Infiltration

**FIGURE 1:**
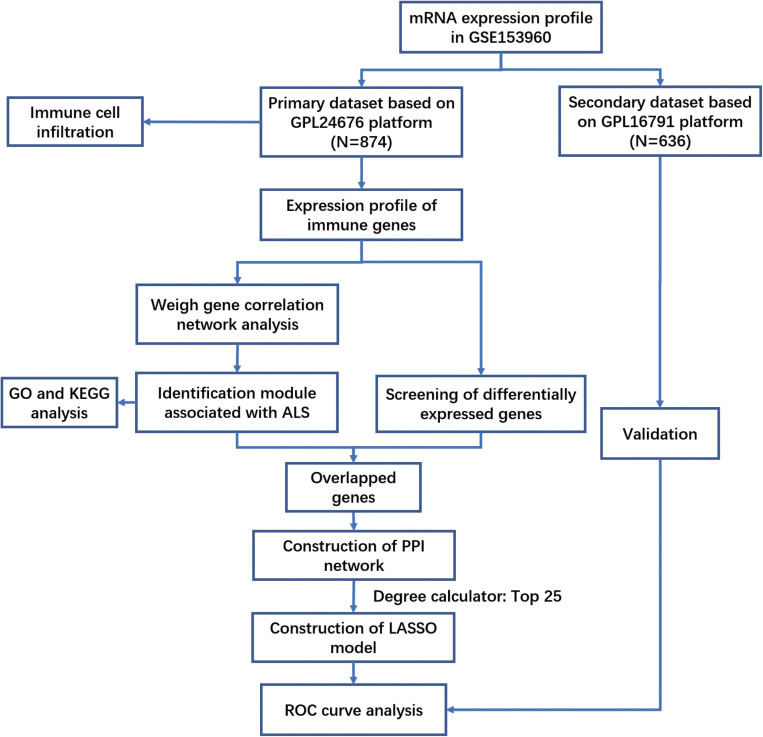
The workflow of this study.

**FIGURE 2:**
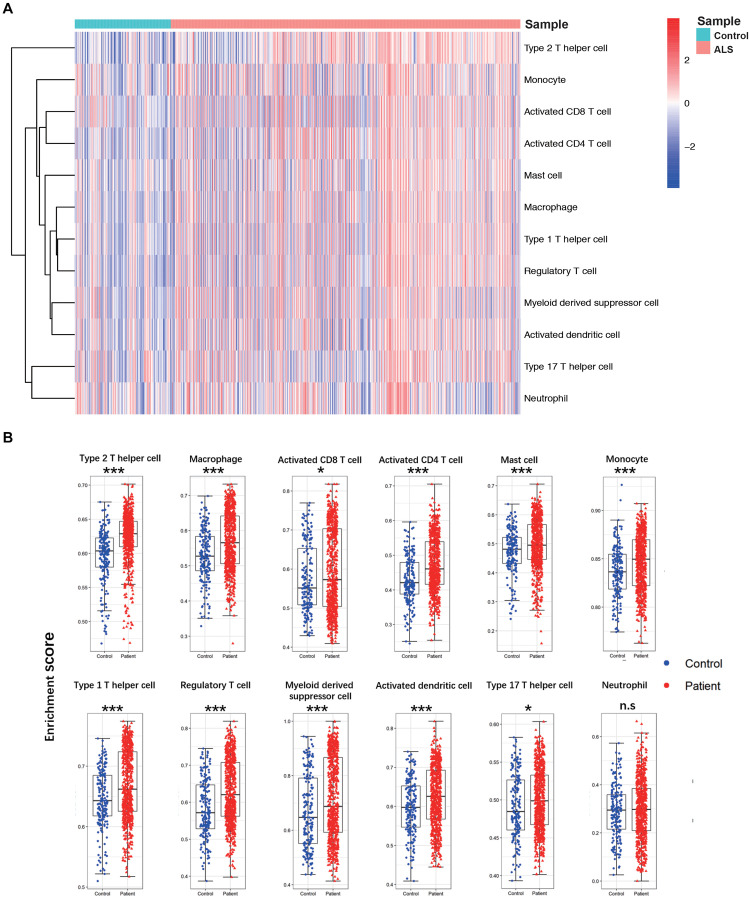
The landscape of immune cell infiltration between ALS patients and non-neurological control subjects. **(A)** A heatmap of 12 immune cell infiltration based on single-sample gene set enrichment analysis. **(B)** Boxplot of comparisons of 12 immune-cell enrichment scores. The enrichment score of macrophages, Tregs, Th1, Th2, Th17, activated CD4 + T cells, activated CD8 + T cells, monocytes, activated dendritic cells, and mast cells myeloid-derived suppressor cells were higher in ALS patients than non-neurological control. *p < 0.05, **p < 0.01, ***p < 0.001.

A flow diagram of the study is shown in Figure 1. The proportions of each tissue type (cerebellum, cortex, spinal cord, and hippocampus) were distributed evenly among the two groups in the primary dataset (ALS vs. controls = 0.09:0.44:0.41:0.06 vs. 0.11:0.49:0.34:0.06; Chi Square-test *p* > 0.05). To investigate differences in immune cell infiltration between ALS patients and non-neurological controls in the primary dataset, we performed ssGSEA analysis of 12 immune cell types. We calculated the enrichment score, which represents the level of immune cell infiltration, and generated a heatmap to visualize the relative abundance of each cell type (Figure 2A). The enrichment scores of most immune cell types were higher in the ALS group than in the control group; 11 immune cell types including macrophages, Tregs, Th1, Th2, Th17, activated CD4 + and CD8 + T cells, monocytes, activated dendritic cells, mast cells, and myeloid-derived suppressor cells showed significant differences in abundance between the two groups (adjusted *p* < 0.05, Wilcoxon test) (Figure 2B), indicating that immune cell infiltration was increased in ALS.

### WGCNA

**FIGURE 3:**
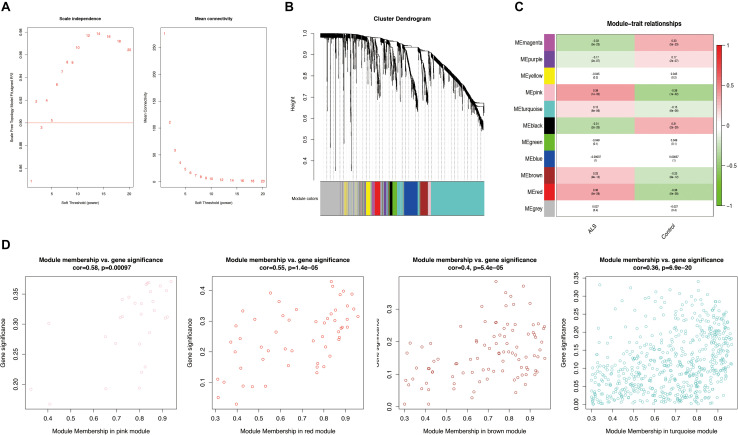
Weighted gene correlation network analysis (WGCNA). **(A)** Analysis of the scale-free network for various soft-thresholding powers (β). A power with the degree of scale independence > 0.9 was considered suitable for constructing the network. **(B)** Identification of co-expression gene modules. The branches of the dendrogram cluster into 11 modules and each one was labeled in a unique color. **(C)** A heatmap showing the correlation between each module eigengene and phenotype. Four modules were positively correlated with ALS-namely, pink, turquoise, brown, and red modules. Red indicates positive correlation and green indicates negative correlation. **(D)** Scatter plot of module eigengenes in the pink, red, brown, and turquoise modules. All these four modules significantly correlated with ALS (*p* < 0.05).

Weighted gene correlation network analysis was performed based on the expression profiles of IRGs in the primary dataset, and a gene co-expression network was constructed. We selected β = 5 as the soft thresholding power (scale-free *R*^2^ > 0.9) to construct a scale-free network (Figure 3A). We then calculated the module eigengenes representing the overall gene expression level of each module; these were clustered based on their correlation. A total of 11 modules were identified and labeled with a unique color (Figure 3B). We analyzed the correlations of each eigengene with phenotype (ALS or non-neurological control) and found four modules were positively correlated with ALS-namely, the pink (cor = 0.39, *p* = 1e-32), red (cor = 0.36, *p* = 5e-28), turquoise (cor = 0.15, *p* = 6e-06), and brown (cor = 0.23, *p* = 9e-12) modules (Figure 3C). Gene significance was calculated to determine the correlation between a gene and phenotype. In our study, a strong association between gene significance and module membership was observed for all four modules (pink: cor = 0.58, *p* = 0.00097; red: cor = 0.55, *p* = 1.4e-05; turquoise: cor = 0.36, *p* = 6.9e-20; brown: cor = 0.4, *p* = 5.4e-05) (Figure 3D). The 783 IRGs in these modules-which are potentially associated with ALS-were retained for further analysis.

### GO and KEGG Pathway Analyses

**FIGURE 4:**
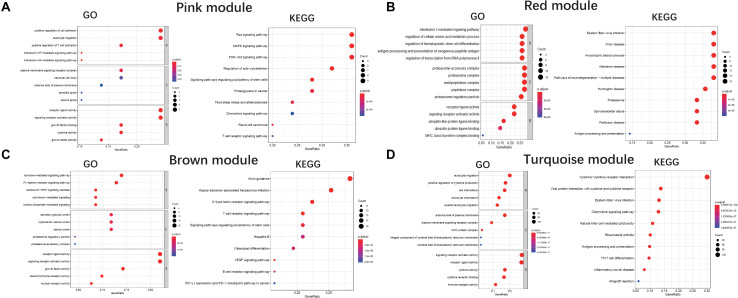
GO and KEGG analysis. Gene Ontology and KEGG enrichment analysis of pink module **(A)**, red module **(B)**, brown module **(C)**, and turquoise module **(D)**, respectively. The color indicates the adjusted *p* value, and the size of dots represents the number of the genes. BP, biological process; CC, cellular component; MF, molecular function.

We performed a GO enrichment analysis of ALS-related modules (i.e., pink, red, brown, and turquoise modules). In the molecular function category, genes in each module were enriched in signaling receptor activator and receptor-ligand activities. The turquoise and pink modules were also related to cytokine activity (Figures 4A–D). The modules were involved in several biological processes including positive regulation of cytokine production (turquoise), antigen processing and presentation (red), positive regulation of T cell activation (pink), and Fc-epsilon receptor signaling (brown) (Figures 4A–D). The KEGG pathway analysis revealed that genes in these modules were mainly enriched in cytokine-cytokine receptor interaction (turquoise), axon guidance (brown), Ras/MAPK/PI3K-AKT signaling pathway (pink), and neurodegeneration including ALS (red) (Figures 4A–D).

### PPI Network Analysis

**FIGURE 5:**
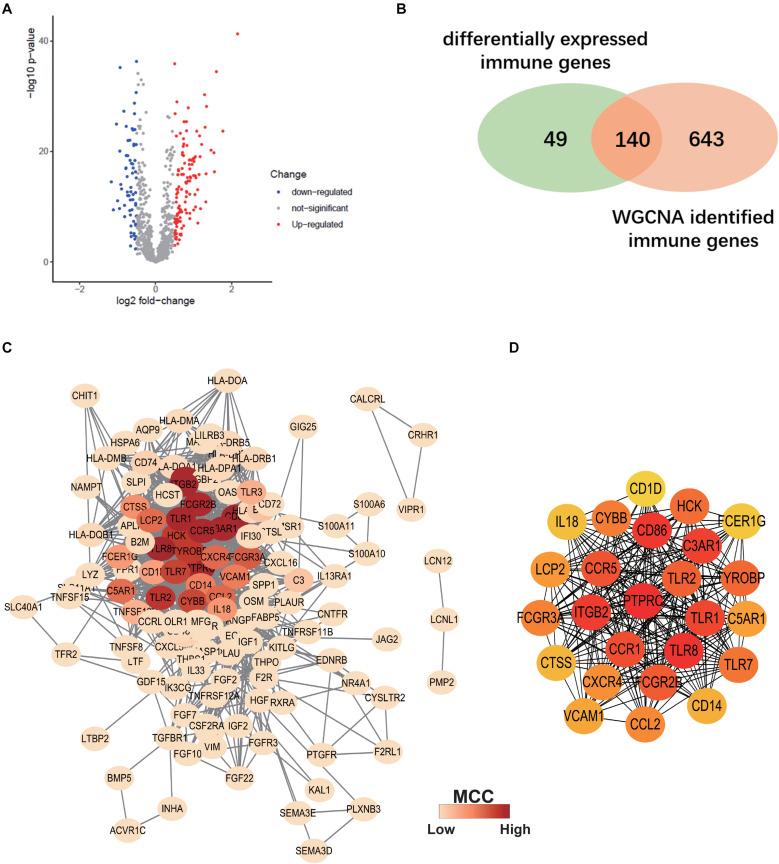
Construction of Protein-protein interaction (PPI) Network. **(A)** Volcano plot of differentially expressed immune-related genes (IRGs) between ALS patients and non-neurological control subjects. Blue dots represent down-regulated genes (66 genes), red dots represent upregulated genes (123 genes), and gray dots represent no significantly differentially expressed genes. **(B)** Venn diagram of overlapping differentially expressed IRGs and ALS-related IRGs derived from WGCNA. **(C)** The PPI network consists of 130 nodes and 1066 edges. Color represents the degree of Maximal Clique Centrality (MCC) calculated by Cytohubba. Red indicates nodes with high MCC score. **(D)** Twenty-five genes with the highest MCC score in this PPI network and their interactions.

Differentially expressed IRGs between ALS patients and non-neurological controls in the primary dataset were evaluated. A total of 189 differentially expressed IRGs were identified with a | log_2_(fold change)| > 0.5 and adjusted *p* < 0.05 (Figure 5A). We overlapped these genes with ALS-related IRGs derived from WGCNA and selected 140 candidate genes (Figure 5B) to construct a PPI network using the STRING online database. Cytoscape software was used to visualize the network, which had 130 nodes and 1066 edges (Figure 5C). To identify the core genes in the network, we used Cytohubba to calculate the MCC of each gene; a high score indicated that the corresponding protein was central to the network. We selected the top 25 genes representing the core genes in the PPI network (Figure 5D).

### Construction and Validation of the LASSO Model

**FIGURE 6:**
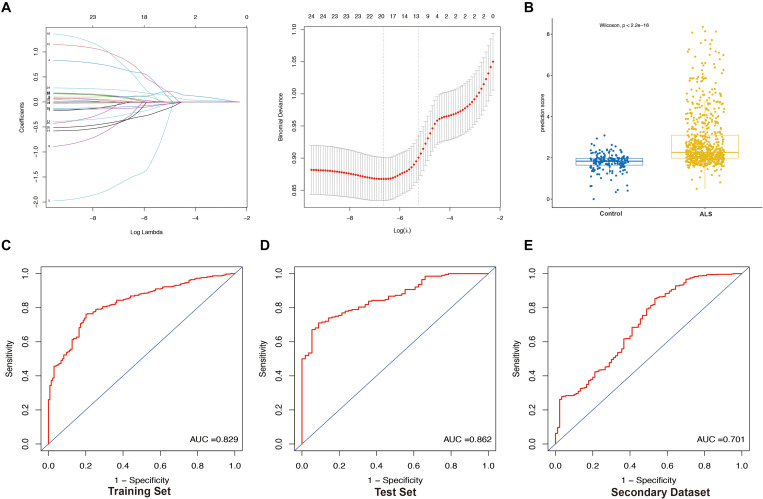
Construction and validation of LASSO model. **(A)** Construction of LASSO model. **(B)** Boxplot was used to visualize the predictive performance of model. Each dot represents the prediction score of each person based on LASSO analysis. Receiver operating characteristic (ROC) curves analysis of predicting the occurrence of ALS. The area under the curve (AUC) was 0.829 in the training set (*N* = 614) **(C)**; 0.862 in the test set (*N* = 260) **(D)**; 0.701 in the secondary dataset (*N* = 636) **(E)**.

We extracted the expression profile (FPKM) of the 25 core genes in the PPI network from the primary dataset and performed LASSO regression analysis to identify the optimal linear combination of core genes for predicting the occurrence of ALS (Figure 6A). A total of 19 genes with non-zero coefficients were selected for model construction. The immune- score was calculated as follows: *C3AR1* expression^∗^(−0.01198) + *CCR1* expression^∗^0.63016 + *CCR5* expression^∗^(−1.54909) + *CD86* expression^∗^(−0.30306) + *CYBB* expression^∗^0.01475 + *FCGR2B* expression^∗^0.10849 + *FCGR3A* expression^∗^(−0.09341) + *HCK* expression^∗^(−0.24098) + *ITGB2* expression^∗^(−0.12903) + *PTPRC* expression^∗^(−0.34727) + *TLR1* expression^∗^0.81489 + *TLR2* expression^∗^0.07799 + *TLR7* expression^∗^0.10668 + *TLR8* expression^∗^0.66320 + *TYROBP* expression^∗^0.08415 + *VCAM1* expression^∗^(−0.34966) + *CD14* expression^∗^(−0.01760) + *CTSS* expression ^∗^0.06362 + *FCER1G* expression^∗^0.22061. The prediction score based on LASSO analysis could efficiently distinguish the ALS patients from non-neurological control subjects (*p* < 2.2e-16, Wilcoxon test) (Figure 6B). In the ROC curve analysis, the area under the ROC curve (AUC) of the model based on the 19 IRGs was 0.829 in the training set (Figure 6C) and 0.862 in the test set (Figure 6D). The predictive value of the model was validated in the secondary dataset, with AUC = 0.701 (Figure 6E). These results indicate that the 19-IRG signature may have diagnostic value for ALS.

## Discussion

Amyotrophic lateral sclerosis remains a fatal neurodegenerative disease with no effective treatments. Significant advances have provided notable insights into the disease mechanisms of ALS, including RNA/DNA dysregulation, metabolism dysregulation, mitochondrial dysfunction and oxidative stress, impaired axonal transport, impaired proteostasis, apoptosis, and immune dysregulation (Robberecht and Philips, 2013; Butti and Patten, 2018; Beers and Appel, 2019; Mejzini et al., 2019). However, it is difficult to translate achievements to human clinical trials due to the great genetic and phenotypic heterogeneity between patients and the complex nature of the disease (Mejzini et al., 2019).

Neuroinflammation and immune dysregulation play critical roles in the disease onset and progression of ALS. Activation of astrocytes and microglia, which release inflammatory and potentially neuroprotective factors, is the most prominent pathologic feature of ALS (Lee et al., 2016). Macrophages, T cells, dendritic cells, and mast cells are known to be involved in ALS pathology but the role of other immune cell types is not well understood (McCauley and Baloh, 2019). In our study, the results of the ssGSEA showed that the infiltration of multiple types of immune cell, including macrophages, Th1, Th17, and Tregs, was greater in ALS patients than in controls. Peripherally derived macrophages can replace depleted microglia, which are the primary innate immune cells of the CNS and are implicated in the progression of neurodegenerative diseases (Butovsky and Weiner, 2018). Macrophages participate in the inflammatory cascade and may contribute to neuroinflammation by entering the CNS parenchyma in ALS patients (Beers and Appel, 2019). In a mouse model of ALS, modifying peripheral macrophage populations slowed disease progression and prolonged survival (Chiot et al., 2020). However, the CNS infiltration of peripheral myeloid cells remains low in ALS mice (Chiot et al., 2020). Th1 and Th17 cells have proinflammatory functions; their activation dominates the immunologic milieu of ALS and may be linked to disease severity (Saresella et al., 2013; Jin et al., 2020). The relative sizes of these cell populations contribute to the speed of disease progression. Tregs have a protective effect against motor neuron death and suppress both proinflammatory Th1/Th17 cells and activated macrophages in ALS (Beers and Appel, 2019; Jin et al., 2020). Stimulation of Tregs populations was shown to have a beneficial effect in ALS patients (Kwon et al., 2014). Autologous administration of the expanded Tregs may slow the disease progression (Fournier et al., 2018). Here, the elevation of Tregs may be explained by responding to increased inflammatory stimulation. The elevated infiltration level in activated CD4 T cell, activated CD8 T cell, Monocyte, and Mast cells were also observed in our research, consistent with previous reports (Rentzos et al., 2012; Murdock et al., 2017; Trias et al., 2018). Taken together, these data suggests that immune activation is a feature of ALS and that therapeutic strategies targeting this process may be an effective treatment.

We performed WGCNA to identify IRGs that contribute to ALS onset or progression. Four modules were positively associated with ALS. The GO analysis revealed that molecular function of receptors and cytokine were the most influenced. Receptors or ligands located on the cell surface influence the activity of the receptor and are involved in signal transduction, both of which participate in immune response (Zhu et al., 2011). Cytokines mediate nuclear signal transduction neurogenesis, neurotransmission, inflammatory responses, and synaptic plasticity (Watkins et al., 1995; Xu et al., 2016). Inflammatory cytokines, released by immune cells such as astrocytes and microglia, can modulate a broad spectrum of cellular responses that are implicated in ALS and other neurodegenerative diseases (Colonna and Butovsky, 2017; Beers and Appel, 2019). Changes in the balance of pro- and anti-inflammatory cytokines have been reported in ALS patients (McCauley and Baloh, 2019). Concerning biological processes, ALS-associated genes were found to be involved in cytokine production, T cell activation, antigen processing and presentation, and Fc-epsilon receptor signaling, indicating the inflammatory cytokines, pathogen recognition, and immune activation are implicated in the pathology of ALS. The most highly enriched pathway in the turquoise module was cytokine-cytokine receptor interaction, which regulates cytokine binding. This pathway is regarded as a crucial aspect of inflammation and is enriched in neurodegenerative diseases such as Alzheimer’s disease (AD), Parkinson’s disease (PD), Huntington disease, multiple sclerosis, and schizophrenia (Guo et al., 2014; Sattlecker et al., 2016; Xu et al., 2016; Khayer et al., 2020). Notably, Ras, MAPK, and PI3K/AKT signaling were among the top enriched pathways in the pink module. Ras acts upstream of MAPK/ERK and PI3K/AKT signaling pathways (Lu et al., 2016). The PI3K/AKT pathway regulates neuronal cell survival and MAPK regulates neural cell proliferation/survival and differentiation, underscoring their potential involvement in the pathogenesis of neurodegenerative diseases (Rai et al., 2019). A recent study showed that inhibiting the PI3K/AKT and MAPK/ERK signaling pathways improved motor activity and survival in transgenic flies expressing mutant hSOD1 (Wang et al., 2018). Thus, drugs that target the PI3K-AKT and MAPK-ERK signaling pathways may have therapeutic benefits for ALS patients. Genes in the red module were enriched in the pathway of ALS, confirming the crucial roles of immune genes in ALS.

We identified 19 immune-related genes (*C3AR1*, *CCR1*, *CCR5*, *CD86*, *CYBB*, *FCGR2B*, *FCGR3A*, *HCK*, *ITGB2*, *PTPRC*, *TLR1*, *TLR2*, *TLR7*, *TLR8*, *TYROBP*, *VCAM1*, *CD14*, *CTSS*, and *FCER1G*) as hub genes in the LASSO regression analysis. This IRG signature could distinguish ALS patients from non-neurological control subjects and predicted the occurrence of ALS. CD86 is a cell surface marker of pro-inflammatory macrophages. In AD patients, CD86 expression increases with age (Busse et al., 2015); the proportion of CD86 + microglia increased with disease progression in SOD1^*G*93*A*^ mice (Hirano et al., 2013). CYBB, also known as NOX2, has been implicated in oxidative stress in neurodegenerative disorders (Cahill-Smith and Li, 2014). In ALS patients, high NOX2 activity was shown to be associated with decreased 1-year survival from onset, indicating that NOX2 could be an independent prognostic factor (Marrali et al., 2014). Thus, NOX2 might be a biomarker of disease severity and hold therapeutic potential for ALS and other neurodegenerative diseases (Sorce et al., 2017). ITGB2 is expressed by microglia/macrophage cells, which are highly associated with ALS progression. Upregulation of ITGB2 expression was observed in the early stage of ALS (Andrés-Benito et al., 2017). A reported gene signature that included ITGB2 was able to differentiate patients according to disease severity (Cooper-Knock et al., 2017). TLR2 was upregulated in reactive glia in the spinal cord of ALS patients, implying that TLR/RAGE signaling was activated, which could promote the progression of inflammation and cause motor neuron injury (Casula et al., 2011). *TYROBP* gene variants have been identified in ALS (Giannoccaro et al., 2017). VCAM1 is a potential marker of preclinical AD and its expression was found to be correlated with disease severity in PD (Andersson et al., 2019; Perner et al., 2019). VCAM1 protein was also upregulated in ALS patients; this may activate the blood-nerve barrier, allowing the entry of circulating inflammatory cells into the peripheral nervous system (Shimizu et al., 2014). Significantly increased expression of CTSS was found in the anterior lumbar spinal cord in ALS cases compared to control subjects (Berjaoui et al., 2015). Although the *C3AR1*, *CCR1*, *CCR5*, and *FCER1G* genes have not been reported in relation to ALS, they have been linked to AD (Liu et al., 2014; Litvinchuk et al., 2018; Sierksma et al., 2020; Wojta et al., 2020), implying a role in neurodegeneration. The *FCGR3A*, *HCK*, and *FCGR2B* genes also offer new directions for investigations on the molecular mechanisms of ALS.

There were some limitations to this study. Firstly, because of a lack of patient information such as age, age of disease onset, disease severity, and phenotype, we were unable to evaluate associations between immune cell populations and disease progression. Secondly, the gene expression data were derived from postmortem brain or spinal cord tissue, which is difficult to obtain in clinical practice. Thirdly, the ALS gene signature in our study was established through *in silico* methods; experimental and clinical data are needed to validate our findings.

## Conclusion

The results of our study demonstrate that immune cells were abundant in the brain and spinal cord of ALS patients, suggesting that they can serve as therapeutic targets. We identified 19 IRGs that are closely associated with ALS and can differentiate ALS patients from controls, and are thus potential biomarkers for disease diagnosis and therapeutic monitoring.

## Data Availability Statement

Publicly available datasets were analyzed in this study. This data can be found here: https://www.ncbi.nlm.nih.gov/geo/.

## Author Contributions

MT and HH designed the study and revised the manuscript. YX and XL analyzed the data. YX provided the first draft of the manuscript. All authors reviewed and approved the final manuscript.

## Conflict of Interest

The authors declare that the research was conducted in the absence of any commercial or financial relationships that could be construed as a potential conflict of interest.

## Footnotes

1 https://www.ncbi.nlm.nih.gov/geo/

2 https://immport.niaid.nih.gov

3 https://www.r-project.org/

4 https://string-db.org/

5 https://CRAN.R-project.org/package=glmnet
